# Using BossDB Tools to Access, Visualize, and Share Volumetric Neuroscience Data

**DOI:** 10.1002/cpz1.70247

**Published:** 2025-10-28

**Authors:** Hannah Martinez, Nicole Guittari, Timothy Gion, Robert Hider Jr, Erik C. Johnson, Jordan Matelsky, Nicole Tregoning, Daniel Xenes, Brock Wester

**Affiliations:** ^1^ Johns Hopkins Applied Physics Laboratory Laurel Maryland

**Keywords:** BossDB, connectomics, electron microscopy, neuroscience, neuroglancer

## Abstract

BossDB is a free and publicly accessible archive for storing and sharing petascale neuroimaging data. Focused on FAIR (i.e., findable, accessible, interoperable, and reusable) principles, it utilizes cloud‐based infrastructure and software tools to facilitate data access and analysis. BossDB specializes in storing volumetric electron microscopy (EM) and X‐ray microtomography (XRM) imaging data along with associated segmentations, annotations, meshes, and connectomes. Users can browse, access, download, visualize, analyze, and upload data through a variety of interfaces, including the BossDB website, Python software development kit (SDK), and web application programming interface (API). Here we present step‐by‐step protocols for using these interfaces and BossDB tools to perform each of these tasks. These protocols target any researcher who is interested in learning more about BossDB public datasets, analyzing high‐resolution neuroimaging and connectomics data with software tools, or contributing a project to BossDB's catalog of public and private data. © 2025 The Johns Hopkins University Applied Physics Laboratory LLC. *Current Protocols* published by Wiley Periodicals LLC.

**Support Protocol 1**: Browsing public data online

**Basic Protocol 1**: Accessing data with Python

**Basic Protocol 2**: Accessing data with data API

**Basic Protocol 3**: Metadata querying via metadata API

**Basic Protocol 4**: Creating a Neuroglancer visualization

**Support Protocol 2**: Creating a BossDB account

**Basic Protocol 5**: Uploading data and metadata

**Basic Protocol 6**: Uploading a small dataset for private use

## INTRODUCTION

BossDB, or the Brain Observatory Storage Service & Database, is a cloud‐based data archive that was initially established in 2018 to meet the open data needs of the connectomics and high‐resolution neuroimaging communities (Hider et al., 2022). Its scalable architecture and interfaces were specifically designed to accommodate increasingly larger image volumes being generated from advancements in electron microscopy (EM) and x‐ray microtomography (XRM) (Dorkenwald et al., 2024; The MICrONS Consortium et al., 2025; Turner et al., 2022). BossDB is a community‐backed resource where scientists can share their large, high‐resolution datasets in a manner that is free, publicly available, and adheres to FAIR (findable, accessible, interoperable, and reusable) data standards. We empower scientists to both contribute to BossDB and perform secondary analysis through numerous open‐source software tools. BossDB currently stores >50 data collections and >1 petabyte of images, segmentations, and connectomes from a variety of species, brain regions, and labs across the world, including from the IARPA MICrONS program and other collections generated from the BRAIN Initiative (see Internet Resources).

BossDB's software ecosystem includes a Python software development kit (SDK), data API, metadata API, and numerous browser resources for visualizing data, performing downloads, and exploring metadata, all accessible at https://bossdb.org. Our Python SDK, entitled “intern”, provides a flexible software interface for interacting with images and segmentations programmatically (Matelsky et al., 2021). It enables users to download, upload, and analyze data using an intuitive and well‐documented library of functions. Our data API provides resources for users to perform similar tasks without the burden of installing any additional software. Our metadata API exposes programmatic access and complex queries of metadata. Finally, our various browser‐based resources enable exploration and analysis through responsive tools connected directly to BossDB data, including visualization of multidimensional data in Neuroglancer.

In this article we will outline detailed process steps of example tasks for use of each of these resources. We will focus on interacting with BossDB‐hosted images, segmentations, annotations, and metadata. BossDB also hosts detailed synapse‐level neural connectivity maps, called “connectomes”, as well as a variety of related tools to search and analyze patterns of connectivity; these capabilities are outlined in a complementary Current Protocols article. First, in Support Protocol 1 we direct you to the Projects page on the main BossDB website, which serves as a browser for all public community‐submitted data. Basic Protocols 1 to 3 show how to access data and associated metadata for these public data collections using the information found on the Projects page. Next, Basic Protocol 4 shows how to visualize multidimensional BossDB data using Neuroglancer (Maitin‐Shepard, 2021). Finally, Support Protocol 2 and Basic Protocols 5 and 6 show how to upload data to BossDB and share it with others.

## Support Protocol 1 BROWSING PUBLIC DATA ONLINE

**Figure 1:**
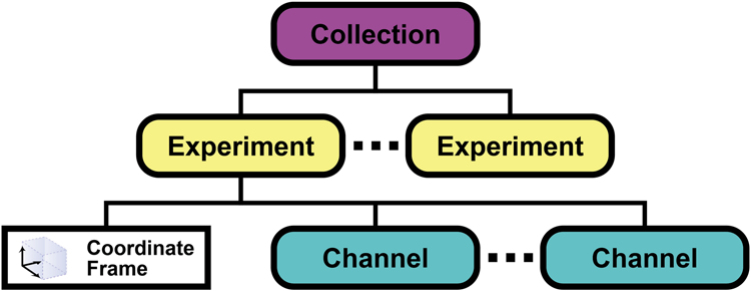
The BossDB data model structure. Data and metadata are organized hierarchically, with “Projects” at the top level, followed by “Collections”, “Experiments” (which define the coordinate frame), and “Channels”.

In this protocol, we show how a user can browse publicly available data projects on https://bossdb.org and view the corresponding image data and metadata. As outlined in a reference page on our website for our data model (https://metadata.bossdb.org/DataModel), each BossDB project is usually associated with a published scientific study and may point to one or more BossDB “data collections”. Each BossDB “data collection” contains one or more “experiments”, where each experiment is generally associated with an individual image volume data acquisition effort that contains a single multidimensional coordinate frame (Fig. 1). Each experiment can have multiple data “channels” all assigned to the same coordinate frame of the experiment. These channels each contain a single data type (e.g., uint8) and can consist of multidimensional image data, segmentation overlays, spatial annotations, and other data spatially registered to the coordinate frame. Together, these components make up a unified project hierarchy. Accessing a BossDB project page is a prerequisite for many other data access and use protocols because each project page includes specific information, such as data Uniform Resource Identifiers (URIs), data organizational (within the BossDB data model), Neuroglancer links, and conveniently individualized code snippets.

### Materials

Computer with internet access
Web browser

1 Visit https://bossdb.org in a web browser.
2 Click the button titled “Browse Public Data”.
3 View data projects and optionally filter them based on study or data collection specific characteristics using the left‐hand options. Choose a data project by clicking its title or cover image.
4 The webpage for the chosen BossDB project will load. On this webpage, browse through the project description, publication link, available data, and more.
5 (Optional) Visualize and explore the multidimensional image, segmentation, and mesh data channels for a project by clicking “View on Neuroglancer”.

## Basic Protocol 1 ACCESSING DATA WITH PYTHON

BossDB provides intern, a Python SDK, for accomplishing a variety of data access and analysis tasks (Matelsky et al., 2021). All users are granted read‐only access to public data regardless of whether they have registered an account with BossDB. In this protocol, we show how to use intern to access images. After completing this protocol, we recommend visiting the BossDB Cookbook and the intern documentation (see Internet Resources) for more examples and to see additional parameters that intern provides for accessing both images and segmentations at multiple resolutions and with different data formats.

### Materials

Computer with internet access
Python ≥3.8, with packages matplotlib and intern installed
Beginner‐level experience with Python

**Figure 2:**
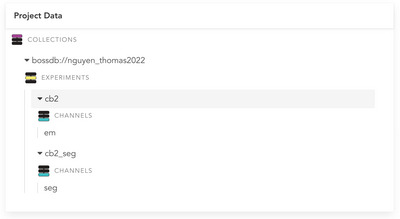
Project Data card for Nguyen et al. (2022), located on its project page. Project Data cards list all collections, experiments, and channels associated with a dataset. Collections, experiments, and channels are organized hierarchically.

1 Follow Support Protocol 1 to navigate to the BossDB project page of interest. For the remainder of this protocol, we will use the Nguyen et al. (2022) project as an example (project page is located at https://doi.org/10.60533/BOSS‐2022‐9RSJ).
2 Within the “Project Data” card on the bottom left of the project page, browse available data channels and choose one to download (Fig. 2). Record the collection ID, experiment ID, and channel ID of the chosen channel. For this protocol, we will choose collection ID “nguyen_thomas2022”, experiment ID “cb2”, and channel ID “em” as an example.
  Note that IDs are case sensitive.

3 Using your method of choice to run Python code (e.g., script, notebook, interpreter, etc.), run the following lines of Python to create an object that can be used to access data. Fill in the collection ID, experiment ID, and channel ID chosen in step 2.


from intern import array
collection_id = "nguyen_thomas2022"
experiment_id = "cb2"
channel_id = "em"
em = array(f"bossdb://{collection_id}/{experiment_id}/{channel_id}")

4 Next, we will download a subset of data using intern and visualize it using matplotlib. When orienting to a new dataset, it is recommended to start in the middle of the volume, as many volumes have empty or black regions at their edges. To calculate the exact middle of a volume, use the following code snippet.
  Notice that intern orders coordinates as ZYX, not XYZ.

shape = em.shape
z_center = shape[0] / 2
y_center = shape[1] / 2
x_center = shape[2] / 2

  Then, a subset of the volume can be downloaded. This code snippet accesses a subvolume that is 1 voxel thick, 1000 voxels wide, and 1000 voxels long.


z_start = z_center
z_stop = z_center + 1
y_start = y_center
y_stop = y_center + 1000
x_start = x_center
x_stop = x_center + 1000
em_subset = em[z_start:z_stop, y_start:y_stop, x_start:x_stop]

  Finally, the subset can be visualized.


import matplotlib.pyplot as plt
plt.imshow(em_subset, cmap="gray")
plt.show()

5 Next, save the image to the file system.


plt.imsave("cutout_test.jpeg", em_subset, cmap="gray")

**Figure 3:**
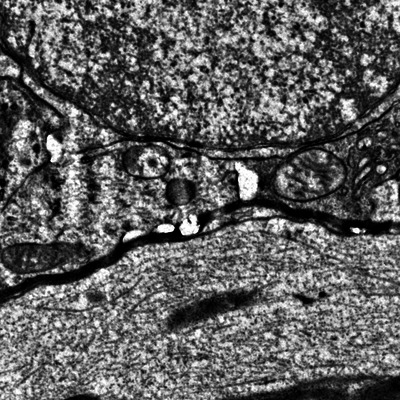
The expected contents of cutout_test.jpeg after completing Basic Protocol 1: Accessing data with Python.

  To find the image's save location, use the operating system's file explorer to navigate to the same location you are running the Python code from, then use your preferred image viewing software to open the file cutout_test.jpeg (Fig. 3). If you are using a script or notebook, this will be the same location where the script or notebook is located. If you are using the Python interpreter or are unsure of where your script or notebook is located, the following Python code will print its location.


import os
print(os.getcwd())

6 For more examples of using intern to access BossDB data using Python, including examples of downloading segmentation, accessing downsampled resolution levels, and more, visit the BossDB Cookbook at https://github.com/aplbrain/bossdb_cookbook and the intern documentation at https://github.com/jhuapl‐boss/intern/wiki (see Internet Resources).

## Basic Protocol 2 ACCESSING DATA WITH DATA API

BossDB provides a web‐based API for performing a variety of data retrieval and management tasks. All users are automatically granted read‐only access to public data regardless of whether they have registered an account with BossDB. In this protocol we show how to use the data API to download images. After completing this protocol, we recommend visiting the Data API Documentation (see Internet Resources) to explore our whole API interface, which includes endpoints for uploading data, tiling and downsampling data, listing all segmentation IDs within a given bounding box, and more.

### Materials

Computer with internet access
Client Uniform Resource Locator (cURL); this comes pre‐installed on the vast majority of operating systems

**Figure 4:**
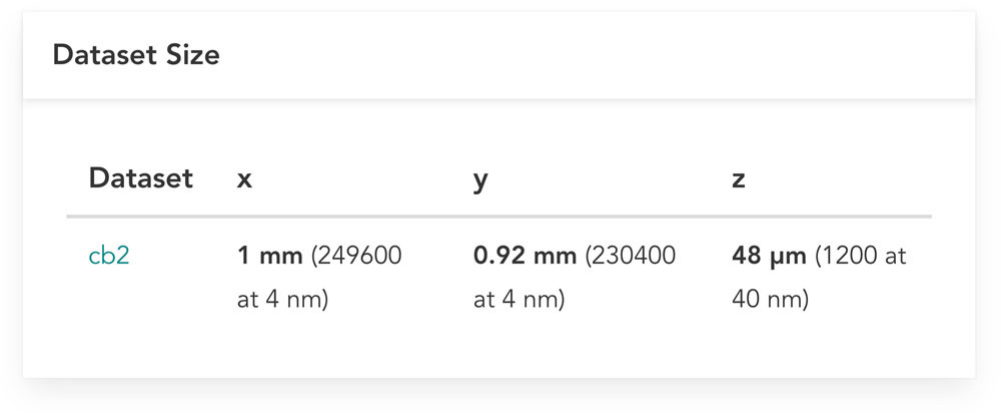
Dataset size card for Nguyen et al. (2022), located on its project page. Shows the resolution and X,Y, and Z extents.

1 Follow Support Protocol 1 to navigate to the project page for a BossDB project of interest. For the remainder of this protocol, we will use Nguyen et al. (2022) as an example (project page is located at https://doi.org/10.60533/BOSS‐2022‐9RSJ).
2 Within the “Project Data” card on the webpage, browse available data channels and choose one to download (Fig. 2). Record the collection ID, experiment ID, and channel ID of the chosen channel. For this example, we will choose collection ID “nguyen_thomas2022”, experiment ID “cb2”, and channel ID “em”.
3 Within the “Dataset Size” card on the webpage, browse the maximum X, Y, and Z coordinates (i.e., data volume extents) for the available BossDB experiments associated with this BossDB project (Fig. 4). Choose a coordinate range in each dimension to download.
  It is recommended to choose coordinates in the middle of the volume, as many volumes have empty regions at their edges. Thus, for this example, we choose a coordinate range close to the center of the example volume (Table 1).

**Table 1:** Coordinate ranges used in Basic Protocol 2 to download a cutout of data

Coordinate direction	Start	Stop
X	123000	124000
Y	111000	112000
Z	600	601

4 Open a terminal application of your choice. On Windows we recommend Powershell, and on MacOS and Linux we recommend Terminal.
5 Copy and run the following multiline command to download a JPEG image of the chosen dataset with the chosen coordinates to the computer's Downloads folder. Be sure to include the authorization header with the BossDB public token.


curl –request GET \
–url 'https://api.bossdb.io/v1/cutout/nguyen_thomas2022/cb2/em/0/123000:124000/111000:112000/600:601?iso=iso' \
–header 'Accept: image/jpeg' \
–header 'Authorization: Token public' \
–output $HOME/Downloads/cutout_test.jpeg

**Figure 5:**
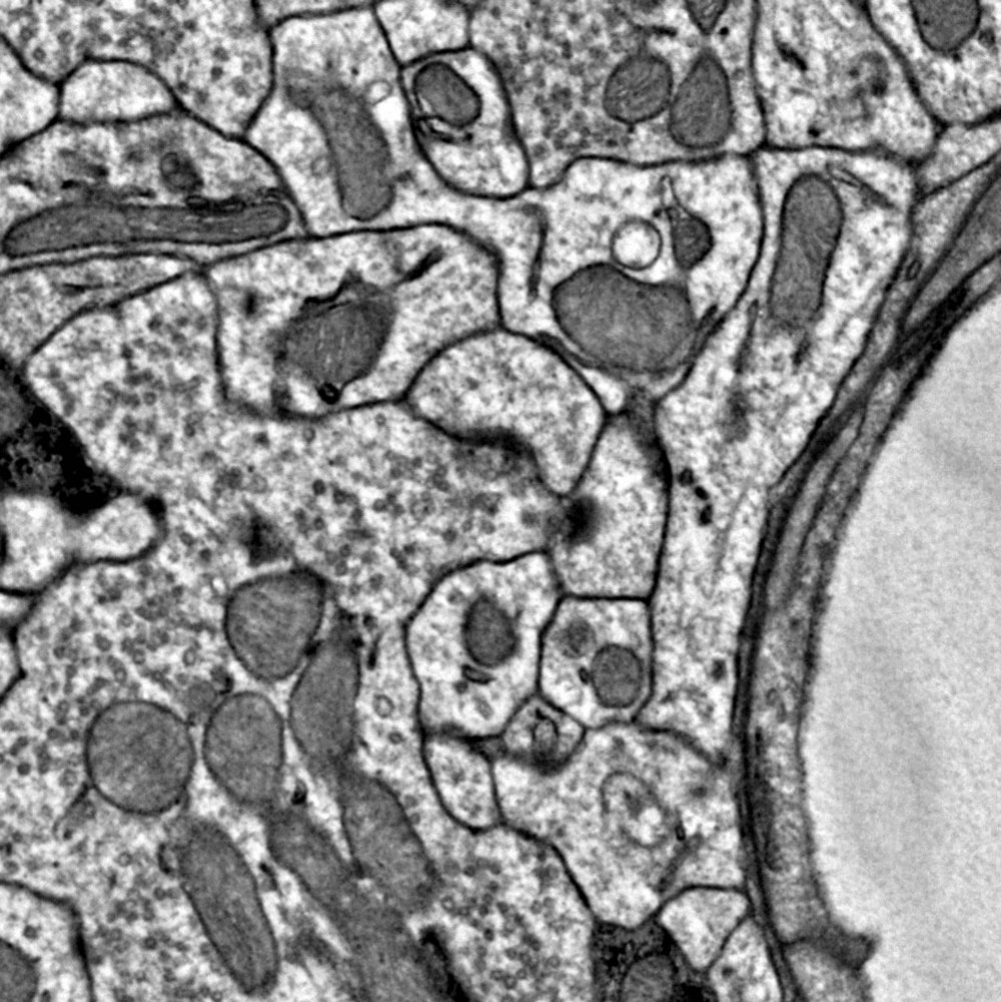
The expected contents of cutout_test.jpeg after completing Basic Protocol 2: Accessing data with API.

  If the command fails because the URL is split onto two terminal lines, try rearranging so that the URL is all on one line.
6 To view the image, navigate to the Downloads folder (or to the path where the data was downloaded) and use your preferred image viewing software to open the file cutout_test.jpeg (Fig. 5).

7 To customize step 5 for your own use case, replace the dataset‐ and coordinate‐specific fields within the second line of the cURL command with your dataset's collection ID, experiment ID, channel ID, x‐range, y‐range, and z‐range.
8 To view other options for this API endpoint, visit the “API Docs” tab at the top of the BossDB webpage.
  The API documentation provides code snippets for repeating this protocol using other platforms such as Python and Node, as well as the option to download to other image formats, including numpy arrays and blosc arrays. Navigate to https://bossdb.org and choose “API Docs” in the navigation menu. Choose Download Cutout in the left menu to view the documentation for the API endpoint used in this protocol.

## Basic Protocol 3 METADATA QUERYING VIA METADATA API

The metadata API, which is a separate resource from the data API referenced above, provides programmatic access to metadata for registered projects, collections, experiments, and channels. Metadata in BossDB is organized hierarchically in a centralized database as seen in Figure 1: a project serves as the top‐level entry; collections group related experiments; an experiment defines a dataset and its coordinate frame; and channels store volumetric data, such as raw images, segmentations, or annotations. Using REST endpoints, users can retrieve records, apply filters, and query subsets of interest for analysis. Results are returned in JSON format and can be integrated into workflows for visualization, benchmarking, or downstream analysis. The metadata API is commonly accessed through Python, as demonstrated in this protocol, but the same queries can also be performed using JavaScript or command‐line tools such as cURL.

### Materials

Computer with internet access
Python ≥3.8, with requests package installed
JavaScript environment (optional)
cURL (optional)

1 Begin at the BossDB Metadata website https://metadata.bossdb.org. From the top navigation menu, click the “API” tab to open the interactive documentation.
  Alternatively, documentation related to the metadata API can be accessed directly at https://api.metadata.bossdb.org/docs, which lists all available routes and allows queries to be tested.
2 Retrieve metadata for a specific experiment by ID. Each experiment in BossDB has a unique object ID, which is different from the human‐readable path (e.g., bossdb://coll/exp). This ID can be found in the experiment's metadata page URL.


import requests
experiment_id = "EXPERIMENT_ID"
url = f"https://api.metadata.bossdb.org/api/latest/experiments/id/{experiment_id}"
response = requests.get(url)
print(response.json())

3 Query channels by type. Common channel types include Image, Segmentation, and Annotation.
  For example, the code below outlines how to list channels labeled as annotations.


query = {"ChannelType": "Annotation"}
url = "https://api.metadata.bossdb.org/api/latest/channels/query"
response = requests.post(url, json=query)
print(response.json())

4 Retrieve schema definitions for collections, experiments, or channels to check required fields.


url = "https://api.metadata.bossdb.org/api/latest/collections/schema"response = requests.get(url)
print(response.json())

5 Perform a more advanced query combining multiple filters.
  For example, the code below outlines how to retrieve all channels within a given experiment that are of type “Segmentation” and contain a specific keyword (e.g., “synapse”).


query = {
"ChannelType": "Segmentation",
"annotations.keyword": "synapse",
"experiment": "EXPERIMENT_ID"
}
url = "https://api.metadata.bossdb.org/api/latest/channels/query"response = requests.post(url, json=query)
print(response.json())

**Figure 6:**
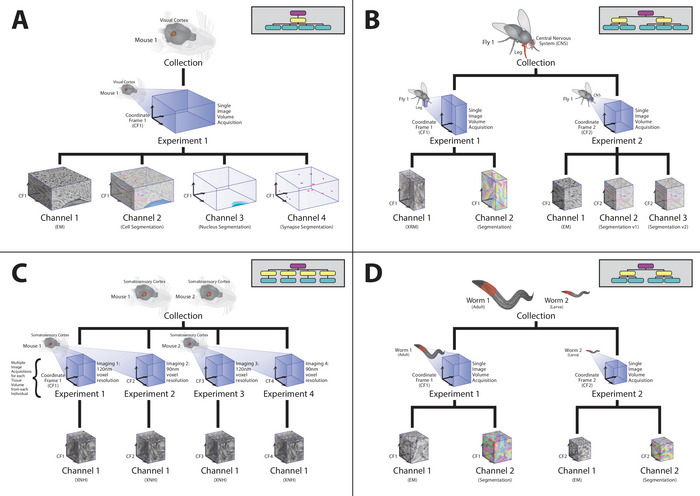
Example dataset hierarchies across species. Data collections contain one or more experiments sharing a coordinate frame, and each experiment contains channels for images and segmentations. (**A**) Mouse visual cortex with one experiment and channels for EM, cell segmentation, nucleus segmentation, and synapse segmentation. (**B**) *Drosophila* CNS with two experiments and channels for EM plus multiple segmentation versions. (**C**) Mouse somatosensory cortex with four experiments imaged at different resolutions, each with an EM channel. (**D**) *C. elegans* with two experiments and channels for EM and segmentation.

  As illustrated in Figure 6, collections may include multiple experiments across species and brain regions, with channels for raw image data and associated segmentations (e.g., cell bodies, nuclei, synapses). Multi‐constraint queries like the above example let you programmatically target just the subset you need (e.g., synapse segmentations in mouse visual cortex).

6 Use the returned JSON output to support metadata‐driven exploration, visualization, or integration into downstream workflows.

## Basic Protocol 4 CREATING A NEUROGLANCER VISUALIZATION

BossDB natively uses Neuroglancer, a web‐based application designed for viewing, exploring, and annotating volumetric data, for visualizing datasets in the browser (Maitin‐Shepard, 2021). Images, segmentations, and annotations are stored in Neuroglancer precomputed format and downsampled several levels to enable excellent load times. In Support Protocol 1, we introduced Neuroglancer by showing that pre‐made visualizations are available on each project page. In this protocol, we will show how to create, customize, and save Neuroglancer visualizations of BossDB‐hosted datasets.

### Materials

Computer with internet access

#### Determine URIs for data to visualize

1 Follow Support Protocol 1 to navigate to the project page for a BossDB project of interest. For the remainder of this protocol, we will use the Park et al. (2023) project as an example (project page is located at https://doi.org/10.60533/BOSS‐2023‐3DLD).
2 A list of volumetric channels is located under the “Project Data” card.
  Channels are organized hierarchically underneath collections and experiments. Channels within the same experiment have the same coordinate frame and can be visualized together in Neuroglancer. Copy the BossDB URI for the channel(s) to visualize in Neuroglancer. A BossDB URI for a channel can be constructed by concatenating the collection, experiment, and channel name together with forward slashes. For example, Park et al. (2023) has image URI bossdb://park2023/cnsl‐cerebellum/em, and segmentation URI bossdb://park2023/cnsl‐cerebellum/seg.

#### Load data into Neuroglancer

3 Open a new browser tab and visit https://neuroglancer.bossdb.io. A blank 4‐panel viewer will load, along with a side panel where a URI can be inputted for visualization.
4 Reformat the BossDB URIs as Data Source URLs that are compatible with Neuroglancer. From each BossDB URI, remove the prefix bossdb:// and add the prefix boss://https://api.bossdb.io/.
5 Load the images into Neuroglancer.
  In the Data Source URL box located in the right panel, enter the newly formatted image URI and hit the ENTER key. A yellow button may pop up with the text “Create as image layer”. Click this yellow button if so. The image data will begin to load chunk by chunk from the underlying BossDB data storage location. Lower resolution image data may load at first; higher resolution image data will successively replace lower resolution data as internet bandwidth allows. By default, BossDB datasets are chunked and downsampled to enable faster load times and accommodate a range of internet communication bandwidths.
6 Load the segmentation into Neuroglancer.
  In the top left of the screen, a tab has appeared titled with the name of the loaded channel. Click the “+” button next to this tab. The “New Layer” panel will reappear on the right side of the screen and allow input for a second Data Source URL. Repeat step 5 with the formatted segmentation URI. The segmentation data should load coincidentally with the registered image data.
7 Use the mouse and keyboard to navigate the visualization. Hold the left‐mouse button to pan; scroll‐wheel to scrub in z; SHIFT + right‐mouse button to rotate; and CTRL + scroll‐wheel to zoom.

#### Annotate in Neuroglancer

**Figure 7:**
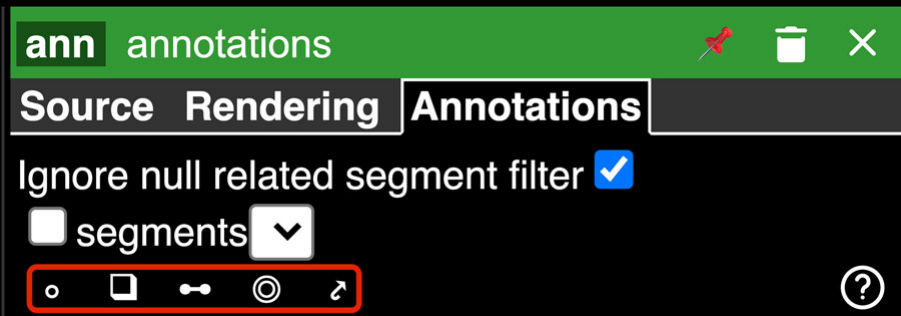
The four annotation tools available in Neuroglancer are highlighted in red: points, bounding boxes, line segments, and ellipsoids.

8 Create an annotation layer.
  In the top left of the screen, there are two tabs titled with the names of the loaded channels. Click the “+” button next to these tabs to reopen the New Layer panel. This time, enter the Data Source URL local://annotations and hit the ENTER key. A yellow button may pop up with the text “Create as annotation layer”. Click it if so. A third tab will appear at the top left next to the tabs for the image and segmentation data, signaling that annotations are ready to be added.
9 Activate an annotation tool. Neuroglancer supports a variety of annotation types, including points, bounding boxes, line segments, and ellipsoids. To add an annotation, click the “Annotations” tab at the top right of the screen, then click one of the icons highlighted in red in Figure 7.

**Figure 8:**
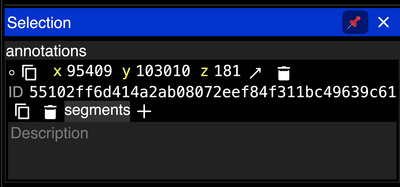
The Neuroglancer selection panel shows information about an annotation, including its XYZ location. The Description box allows a user to add a text label.

10 Add annotations on top of the volumetric data using CTRL + left‐click. For each annotation, the “Selection” panel will appear on the right (Fig. 8). Text labels can be added to an annotation using the “Description” field in its “Selection” panel.

#### Saving a Neuroglancer view

11 To save a Neuroglancer view, copy the URL from the browser address bar and keep it for your records. Neuroglancer uses URL state management, so all parameters for an entire visualization are encoded in the URL. BossDB does not currently track or record your Neuroglancer visualization or annotation states.

## Support Protocol 2 CREATING A BossDB ACCOUNT

Having a BossDB account is a prerequisite for uploading data for personal use and for editing data and metadata associated with data collections, experiments, and channels. In this protocol, we describe how to make a BossDB account and generate an API token for programmatic access to private datasets.

### Materials

Computer with internet access
Email address

1 Navigate to https://bossdb.org in a web browser. Click the “Metadata” tab. Once the metadata website has loaded, click the “Login” button at the top right.
2 A login page will load. Click “Register” at the bottom and fill in the required fields to create a new account.
3 You will be redirected to the Boss Management Console. This website is where private and public datasets are managed by both users and system administrators, respectively.
4 For Basic Protocol 6, you will need an API token. Click your username in the top right corner, then “API Token”. The API Token page will load. Click “Generate Token” to create an API token. Keep this token private, like a password, as anyone with this token can access your private datasets. You can revoke and generate a new token at any time on this page.

## Basic Protocol 5 UPLOADING DATA AND METADATA

The BossDB Metadata Portal provides a guided workflow for contributors to upload datasets along with descriptive metadata. Through this interface, researchers can register their projects with standardized schema, making them discoverable through both the web portal and the API. Metadata capture includes information, such as project title, species, imaging modality, data size, and related publications. Once submitted, the BossDB team validates entries and coordinates data ingestion, ensuring consistency and long‐term accessibility.

### Materials

Computer with internet access
BossDB account (see Support Protocol 2)
Dataset prepared in supported format (e.g., EM volumes, segmentations, annotations)
Values for each metadata field (e.g., species, modality, voxel size, acquisition parameters, channel types)

1 Navigate to https://metadata.bossdb.org in a web browser and log in with your BossDB account. From the navigation menu, click “Upload Data”.
2 Fill in the Basic Information form, including corresponding author and year, project title, and an optional data project description. Choose whether the data will be public or private.
3 Specify the “Estimated Data Size” by selecting either <50 GB (for smaller uploads) or >50 GB (for concierge‐assisted uploads). Click Continue.
4 Select the “Species” and “Imaging Modality” for your data collection(s), choosing from the provided biological specimens and imaging modalities. Click Continue.
5 Provide “Funding & Publications” information, indicating whether the dataset funded by the BRAIN Initiative. Optionally, add grant numbers and related DOIs.
6 Review the summary screen and click “Finalize”. For data that are marked as public or >50 GB in size, a BossDB team member will follow up at the email address provided to coordinate the upload or publicize the privately uploaded data. For datasets <50 GB, proceed to Basic Protocol 6 for instructions on privately uploading data.

## Basic Protocol 6 UPLOADING A SMALL DATASET FOR PRIVATE USE

BossDB stores all public and private data in Amazon Web Services cloud storage. User upload of small volumetric image datasets to cloud storage for personal use with BossDB tools is supported through intern, the BossDB Python SDK previously introduced in Basic Protocol 1. Once data is uploaded to BossDB, users can share them with other users, analyze them with BossDB tools, and publicize them by following Basic Protocol 5. This protocol describes the process for uploading private data to BossDB.

### Materials

Computer with internet access
BossDB API token (can be generated in Support Protocol 2)
Python ≥3.8, with the package intern installed
Beginner‐level experience with Python
Data to upload (e.g., stack of 2D images, single 3D image file or array)
Python package that allows for programmatic access to your image format (some suggestions are shown in Table 2)

**Table 2:** Python packages that allow for programmatic access to images depending on format

Image format	Suggested Python package
PNG, JPEG	PIL
TIFF	tiffile
Zarr	ome‐zarr

*NOTE*: This protocol is meant for datasets <50 GB in size. If your dataset is larger, please follow Basic Protocol 5 and select the option for a dataset >50 GB in size. A BossDB team member will follow up and assist you with a concierge‐style ingest.

1 Configure intern to use your BossDB API token by creating an intern configuration file and placing the API token inside. The intern configuration file should be called intern.cfg and should be placed at C:Users\<myusername>\.intern\intern.cfg on Windows systems and ∼/.intern/intern.cfg on MacOS and Linux systems. Inside this file, fill in the following contents:


[Default]
protocol = https
host = api.bossdb.io
token = <your token here>

  Save and close the file.
2 Construct a new BossDB URI for your dataset. An example of a BossDB URI is bossdb://park2023/cnsl‐cerebellum/em. You can think of it as a way of organizing projects into a three‐tiered directory structure. For a test dataset, a good URI choice would be something like bossdb://<yourusername>/test/images.
3 Using your method of choice to run Python code (e.g., script, notebook, interpreter, etc.), run the following lines of Python to create resources in the BossDB system for your new dataset. Fill in the variables BOSSDB_URI with the URI you created in step 2, SHAPE_ZYX with the length, width, and height of your dataset in voxels, VOXEL_SIZE with the dimensions of a single voxel, and VOXEL_UNITS with the unit that VOXEL_SIZE is in.


from intern import array
BOSSDB_URI = <your URI>
SHAPE_ZYX = <tuple or list denoting dataset shape>
VOXEL_SIZE = <tuple or list denoting dataset voxel size>
VOXEL_UNITS = <string denoting units, e.g., nanometers>
new_boss_dataset = array(
BOSSDB_URI,
extents=SHAPE_ZYX,
voxel_size=VOXEL_SIZE,
voxel_unit=VOXEL_UNITS,
create_new=True
)

4 Load a 2D image using the appropriate Python library. intern's array object behaves very similarly to a numpy array, so manipulating your image into a format compatible with numpy will typically yield good results. A code snippet below shows how to load a TIFF file.


import tifffile
im_array = tifffile.imread('path/to/your/image.tif')

5 Upload the image to the dataset you made in step 3.


slice_number = 0
new_boss_dataset[slice_number,:,:] = im_array

6 Repeat steps 4 and 5 for all images until the whole dataset has been uploaded.
7 To see the uploaded data in Neuroglancer, print a link and paste it into a web browser's address bar.


print(new_boss_dataset.visualize)

## COMMENTARY

### Critical Parameters

Careful attention to a few key factors is necessary for obtaining reliable and reproducible results with BossDB tools. Across all the protocols, accuracy of text and values in metadata entry, coordinate selection, and URI construction is especially important, as small errors (e.g., incorrect case and other typos) in these areas can prevent successful data retrieval or visualization. The following subsections describe the most critical parameters for each protocol in detail.

#### Support Protocol 1: Browsing public data online

The most important factor when browsing BossDB's public datasets is careful attention to details provided in each project page. Each project page contains unique information including URIs, Neuroglancer links, and channel identifiers that are required for subsequent protocols. If users skip this step or copy identifiers incorrectly, subsequent attempts to access data programmatically or via the API will fail.

#### Basic Protocol 1: Accessing data with Python

When using the Python SDK, intern, accuracy of alphanumeric values in specifying the collection, experiment, and channel identifiers is essential. A single mismatch will result in an error or an empty dataset. Another critical parameter is coordinate selection, as many datasets include padded or empty space at their edges. Thus, it is recommended that initial test subvolumes should be chosen near the center of the dataset. Users should also remember that intern uses ZYX ordering, not XYZ, which is a common source of confusion. Stable Python environments with the correct package versions are necessary to avoid dependency conflicts.

#### Basic Protocol 2: Accessing data with data API

When retrieving data through the data API, properly constructed cutout requests determine whether an image loads successfully. Errors most often occur when coordinate ranges extend beyond the dataset bounds or when the collection, experiment, or channel names are mistyped. Users must also include the correct authorization header for private datasets, since even small token errors will block access.

#### Basic Protocol 3: Metadata querying via metadata API

For metadata queries using the metadata API, completeness and accuracy of metadata fields are critical. Submissions that omit required information, such as voxel size, imaging modality, or species, will not pass validation and cannot be registered. Similarly, users querying metadata must ensure they are using the correct schema definitions to avoid failed query requests.

#### Basic Protocol 4: Creating a Neuroglancer visualization

Visualization success depends on properly formatting URIs for Neuroglancer. Users must remove the bossdb:// prefix and replace it with the correct boss:// https://api.bossdb.io/ string. Failure to do so will prevent the dataset from loading. Internet bandwidth is another key parameter, as Neuroglancer streams downsampled data chunks that require stable connections. Slower connections may result in incomplete loading or significant latency.

#### Support Protocol 2: Creating a BossDB account

The critical parameter for account creation is careful handling of API tokens. These tokens grant full access to private data and must be kept confidential. Any misplaced or expired token will prevent further access until replaced, and users should be aware that tokens can be regenerated if compromised.

#### Basic Protocol 5: Uploading data and metadata

Public uploads of new data are sensitive to metadata accuracy and data formatting. Datasets must be correctly structured in supported formats and paired with comprehensive metadata including acquisition parameters and voxel units. Missing or inconsistent metadata will delay ingestion and may require manual correction. Large datasets >50 GB also require coordination with the BossDB team, making planning and communication important parameters for success.

#### Basic Protocol 6: Uploading a small dataset for private use

Private uploads depend heavily on correct URI construction and metadata entry. Users should select logical and unique URIs such as bossdb://username/test/images to avoid conflicts. The dataset's voxel size, shape, and units must be specified correctly at the time of dataset creation since errors here can render the dataset unusable in Neuroglancer or downstream tools. Correct configuration of the intern SDK, including placement of the token in the intern.cfg file, is also essential for reproducible results.

### Troubleshooting

Common problems encountered when using BossDB tools are summarized in Table 3. These issues most often arise from errors in URI construction, coordinate selection, metadata entry, or token configuration.

**Table 3:** Troubleshooting guide for common data access and upload issues with BossDB

Problem	Possible cause	Solution
Blank or empty image after download	Coordinates selected outside dataset bounds or in padded edges	Recheck dataset size on the project page and choose coordinates closer to the dataset center
API request fails with 401 or 403 error	Missing, expired, or misformatted API token	Generate a new token in the BossDB Management Console and confirm correct placement in configuration or request header
Neuroglancer visualization does not load	URI incorrectly formatted (bossdb:// prefix not converted)	Replace prefix with boss:// https://api.bossdb.io/ and verify identifiers match project page
Upload rejected by metadata system	Missing or inconsistent metadata fields, e.g., voxel size, modality, or species	Retrieve schema definitions from the Metadata API and ensure all required fields are complete and accurate
Slow loading or incomplete visualization	Limited internet bandwidth or very large dataset	Use downsampled resolution levels or smaller cutouts and confirm stable internet connection
Private upload fails	intern configuration file missing or misformatted	Confirm that intern.cfg is in the correct location and contains the token, host, and protocol information
Dataset not visible after upload	Incorrect voxel size, shape, or units specified during dataset creation	Recreate the dataset with corrected parameters and verify dimensions before uploading
Commands from the protocols fail when connected to a VPN, including intern Python downloads and cURL cutouts	VPN policy is proxying or blocking outbound HTTPS traffic, preventing requests from reaching external endpoints as expected	Disconnect from VPN or enable split tunneling for external sites or confirm that HTTPS on port 443 is allowed; if needed, ask IT to adjust VPN proxy settings to allow direct access to external resources

### Understanding Results

Successful execution of the BossDB protocols leads to reproducible outputs that can serve as benchmarks for new users. For Support Protocol 1, browsing public data online should result in a clear view of project pages containing dataset descriptions, metadata, publication links, and available channels. A correct outcome is the ability to locate dataset identifiers, URIs, and Neuroglancer links that will be reused in later steps.

In Basic Protocol 1, accessing data with Python should generate a valid image cutout saved as a JPEG or array file. Users should expect image and associated segmentation data of tissue with recognizable structures, such as cell bodies or membranes, depending on the modality. A blank or noisy output suggests that coordinates were mis‐specified. Similarly, Basic Protocol 2 should yield a saved image file when the data API is queried; success is confirmed when the cutout matches the requested dimensions and appears in the Downloads folder without corruption.

Metadata queries through the metadata API in Basic Protocol 3 should return structured JSON records containing project details, species, imaging modality, and other metadata fields. A correct result is a complete and readable response consistent with the schema, which can then be parsed or integrated into downstream workflows.

For Basic Protocol 4, Neuroglancer visualization should load images and, if available, corresponding segmentation and annotation layers. Successful results include smooth scrolling through volumetric planes, visible overlays of segmentation and annotation data, and functional annotation tools. Negative results may include incomplete loading due to bandwidth limitations or misformatted URIs.

Support Protocol 2 is confirmed when an account is created successfully, and an API token is generated. The expected outcome is access to the Boss Management Console and the ability to store the token securely for later use.

In Basic Protocol 5, public dataset upload is successful when metadata validation passes, and the dataset appears in the BossDB catalog. Users should be able to confirm this by navigating to the project page and inspecting the registered metadata. Negative results typically arise from incomplete fields or inconsistent metadata entries.

For Basic Protocol 6, private dataset upload is validated when the dataset can be opened in Neuroglancer via the generated visualization link. A correct result is a dataset that appears with the correct dimensions, voxel size, and units, and is accessible through the Python SDK. If the dataset fails to load, it may indicate that voxel parameters were entered incorrectly during setup.

Across all protocols, expected results can be checked against the figures provided in this article, which illustrate sample cutouts, visualizations, and metadata responses. Users should use these benchmarks to confirm they have correctly reproduced each step.

### Time Considerations

The time required to complete each protocol depends on a combination of dataset size, available compute resources, network bandwidth, and the user's familiarity with the BossDB ecosystem. Support Protocol 1 can usually be completed within ∼5 min once a BossDB project of interest is identified. Protocols involving Python or API access, such as Basic Protocols 1 through 3, may take anywhere from ∼5 min to 0.5 hr depending on whether the user already has the necessary software environment and dependencies in place. Creating Neuroglancer visualizations as described in Basic Protocol 4 can be accomplished quickly for small datasets, but large datasets and limited bandwidth can significantly increase load times.

Support Protocol 2 typically takes only ∼5 min but requires additional time if token configuration is unfamiliar. Uploading data, whether for public or private use (Basic Protocols 5 and 6), is the most variable in terms of time. Small datasets that are already in a supported format can be uploaded in <1 hr, while larger datasets require more extensive preparation, metadata entry, and potentially coordination with the BossDB team. In general, users with established environments and experience in cloud‐based data handling will complete these protocols more quickly than first‐time users who need to set up accounts, dependencies, and workflows from scratch.

### Author Contributions

**Hannah Martinez**: Conceptualization; software; writing—original draft. **Nicole Guittari**: Conceptualization; software; writing—original draft. **Timothy Gion**: Software. **Robert Hider Jr**: Software; writing—review and editing. **Erik Johnson**: Software; writing—review and editing. **Jordan Matelsky**: Software. **Nicole Tregoning**: Software; writing—review and editing. **Daniel Xenes**: Software. **Brock Wester**: Conceptualization; funding acquisition; project administration; supervision; writing—review and editing.

### Conflict of Interest

The authors certify that they have no affiliations or involvement with any organization or entity with any financial interest in the subject matter discussed in this manuscript.

## Data Availability

The data that support the protocols are openly available in BossDB at https://doi.org/10.60533/BOSS‐2022‐9RSJ and https://doi.org/10.60533/BOSS‐2023‐3DLD.
